# Electrocardiographic Abnormalities in Patients With Acute Exacerbation of Chronic Obstructive Pulmonary Disease

**DOI:** 10.7759/cureus.20820

**Published:** 2021-12-30

**Authors:** Wikram Kumar, Wajid Shaikh, Sandeep KP, Shaheen Bhatty, Sadaf Hassan

**Affiliations:** 1 Medicine, Dr. Ruth K. M. Pfau Civil Hospital Karachi, Karachi, PAK; 2 Endocrinology, Diabetes, and Metabolism, Baqai Institute of Diabetology and Endocrinology, Karachi, PAK; 3 Internal Medicine, Dr. Ruth K. M. Pfau Civil Hospital Karachi, Karachi, PAK; 4 Medicine and Surgery, Chandka Medical College Larkana, Larkana, BOL

**Keywords:** electrocardiographic, acute exacerbation, copd, risk factors, prevalence

## Abstract

Objective: The objective of this article was to determine the frequency of different electrocardiographic (ECG) abnormalities in patients with acute exacerbation of chronic obstructive pulmonary disease (COPD).

Design: This is a cross-sectional study.

Place and duration: The study was conducted at the medicine department at Civil Hospital Karachi, Pakistan, between November 2018 and May 2019.

Method: Both female and male participants aged 18-60 years with acute exacerbation of COPD (as per the operational definition) for more than three days who did not receive any treatment for exacerbation were included in the study. Twelve-lead ECG was recorded (Schiller AG, Baar, Switzerland) for 10 minutes after the supine rest, with a 50 mm/s of paper speed, 10 mm/mV of gain, and filter default settings.

Results: In total, 140 participants (male: n = 124 [88.6%] and female: n = 16 [11.4%]) were included the research. The mean age of the participants was 40.43 ± 11.51 years. In terms of severity, 46 (32.95) patients presented with mild, 46 (32.9%) with moderate, and 48 (34.4%) with severe exacerbation. Moreover, 33 (23.65%) participants had ECG abnormalities, i.e., 13 (9.3%) patients presented with right atrial enlargement, and eight (5.7%) with right ventricular hypertrophy. Patients with a longer smoking duration (years) were likely to present with ECG abnormalities.

Conclusion: Patients with COPD who had severe acute exacerbation and a long smoking duration have a high prevalence of ECG abnormalities. Hence, ECG may be a valuable tool for detecting ischemic abnormalities among patients with COPD, independent of previously known heart disease, in clinical settings.

## Introduction

Chronic pulmonary obstructive disease (COPD) is a common, preventable, and treatable disease characterized by chronic poor airflow. Moreover, it is a major global public health issue [1].

COPD ranks fifth in terms of disease burden worldwide and third in terms of mortality worldwide. In the United States, it is currently the leading cause of mortality and the second leading cause of morbidity [2]. Low-middle income countries, including Pakistan, face unique challenges in diagnosing and managing COPD, particularly during exacerbations, due to suboptimal and diverse primary care systems [3].

The prevalence of COPD in Pakistan was approximately 13.8% [4]. Tobacco smoking with a number of other factors is the most common cause of COPD, such as indoor and outdoor air pollution, alpha-1 antitrypsin genetic deficiency (AAT), occupational dust, and frequent chemical exposure [5]. The extrapulmonary manifestations of COPD include cardiovascular diseases, osteoporosis, skeletal muscle dysfunction, metabolic syndrome, depression, and lung cancer [6]. Patients with COPD are at a higher risk for independent cardiovascular morbidity and mortality [7].

COPD is associated with specific electrocardiographic (ECG) abnormalities. Previous studies have reported that patients with COPD had a higher risk of myocardial infarction, cardiac arrhythmia, atrial fibrillation, non-sustained ventricular tachycardia, and sustained ventricular tachycardia [8,9]. International studies about acute COPD exacerbation have revealed that the frequency of a new ECG finding is high during exacerbation compared with baseline. In a recent study, 8%-13% of all patients admitted to hospitals due to acute COPD exacerbation present with atrial fibrillation [10]. Further, another research showed that with COPD, 22%-40% of all patients experience at least one severe or moderate exacerbation annually [11]. Patients experiencing frequent exacerbations present with poor quality of life and accelerated lung function decline, and they are at a higher risk for myocardial infarctions, future exacerbations, events of cerebrovascular accidents, and mortality.

Several ECG abnormalities were observed in patients with stable COPD and those with acute COPD exacerbation. However, local data about acute exacerbations are rare. Hence, the current study aimed to elucidate the frequency of ECG abnormalities in patients with acute COPD exacerbation to improve the interpretation of ECG findings and identify the associated cardiac abnormality and appropriate treatment, thereby reducing the risk of morbidity.

## Materials and methods

This descriptive cross-sectional study was conducted at the department of medicine and the intensive care unit of Dr. Ruth K. M. Pfau Civil Hospital, Karachi, from January 21, 2020, to July 20, 2020. Moreover, it was approved by the institutional review board.

Informed consent was obtained from each participant after explaining the study protocol. The research lasted for six months (between November 2018 and May 2019). The non-probability consecutive sampling technique was used. The sample size was calculated by assessing a 95% confidence interval (1-a). The expected population proportion (P) was 8%, and the absolute precision (d) was 4.5%.

The following formula was used: Z2 x (P) x (1-P)/d2, where Z indicates the Z value (e.g., a 95% confidence interval of 1.96), P is the prevalence of disease, and d is the absolute precision of 5% (0.05).

Both male and female patients aged 18-60 years with acute COPD exacerbation (as per the operational definition) for more than three days who did not receive any treatment were included in the study. Meanwhile, patients with a history of any treatment for acute exacerbation within three days; those without acute exacerbation; those with asthma (ratio of the forced expiratory volume in the first one second to the forced vital capacity of the lungs [FEV1/FVC] of <70%, but improving by 12% after treatment with bronchodilators); and those with a history of cerebrovascular accident (CVA), head or spinal cord trauma, or fractured limbs were excluded from the study. Moreover, patients diagnosed with heart failure, ischemic heart disease, ST elevation, myocardial infarction, rheumatic heart disease, congenital heart disease, or chest trauma were not included.

Acute COPD exacerbation was defined clinically as dyspnea (respiratory rate of >24 cycles/min), along with the presence of at least a single episode of fever (100°F) within the last three days, oxygen saturation of less than 84%, smoking history of >10 pack years, and FEV1/FVC of <70% on spirometry (not improving by 12% after treatment with bronchodilators). FEV1/FVC was categorized as follows: mild, <70% but >60%; moderate, <60% but >50%; and severe <50% [12].

Twelve-lead ECG (Schiller AG, Baar, Switzerland) for 10 minutes were recorded after the rest of supine, with a 50 mm/s paper speed, 10 mm/mV of gain, and default filter settings [13]. The findings were interpreted by the researcher with the supervision of an experienced cardiologist. The ECG abnormalities were right atrial enlargement (P wave amplitude in leads II, II, and augmented vector foot [aVF] of >2.5 mm or V1 of 21.5 mm), right ventricular hypertrophy (R in V1: >7 mm; R/S in V1: >1; or ventricular activation time in V1: >35 ms), clockwise rotation (R/S ratio in V5: <1), low voltage limb leads (QRS [R+S]: <5 mm in I, II, aVF, and III), QS complex (if present in lead III), right axis deviation (>90°), left axis deviation (<−30° to −90°), prolonged QT interval (>0.44 s), atrial fibrillation (>three sawtooth waves in between two QRS complexes in lead II), T wave changes (>1 mm depression below or >5 mm elevation above baseline), and ST depression (>1.5 mm below baseline). All values were catered to in the pre-approved proforma.

## Results

A total of 140 subjects were included: 124 (88.6%) males and 16 (11.4%) females. The number of female patients was less as females in our population tend to smoke less. The mean age of subjects was 40.43 ± 11.51 years. The mean duration of smoking was 6.42 ± 1.60 years with the mean pack used per year as 13.64 ± 1.76 packs. The severity of exacerbation shows that 46 (32.95) subjects had mild, 46 (32.9%) had moderate, and 48 (34.4%) had a severe exacerbation. The mean oxygen saturation for subjects was 75.87 ± 3.378 (Table 1).

**Table 1:** Baseline characteristics of the study participants Data are presented as mean ± SD/n (%). FEV1/FVC, Ratio of the forced expiratory volume in the first one second to the forced vital capacity of the lungs.

Characteristics	Mean ± SD/n (%)
N	140
Male	124 (88.6%)
Female	16 (11.4%)
Age	40.43 ± 11.51
Duration of smoking (years)	6.42 ± 1.605
Cigarette packs used per year	13.64 ± 1.76
FEV1/FVC	
Severe	46 (32.9%)
Moderate	46 (32.9%)
Mild	48 (34.3%)
Oxygen saturation	75.87 ± 3.378

Abnormal ECG changes were seen in 33 (23.65%) subjects. While comparing the types of ECG changes, right atrial enlargement was found higher in 13 (9.3%) subjects followed by right ventricular hypertrophy in eight (5.7%), right axis deviation in five (3.6%), low axis deviation in three (2.1%), clockwise rotation in three (2.1%), and low voltage limb leads in one (0.7%) (Table 2).

**Table 2:** ECG changes and the types of ECG changes present Data are presented as n (%).

ECG Changes	n (%)
Yes	33 (23.6%)
No	107 (76.4%)
Types of ECG changes present	
Right atrial enlargement	13 (9.3%)
Right ventricular hypertrophy	8 (5.7%)
Right axis deviation	5 (3.6%)
Low axis deviation	3 (2.1%)
Clockwise rotation	3 (2.1%)
Low voltage limb leads	1 (0.7%)

ECG changes with no significant difference were higher in the younger age group in 13 (39.4%) subjects as compared to the middle and older age groups. No significant difference was also seen for ECG changes among males and females. Subjects with a higher duration of smoking (years) had a significantly elevated frequency of ECG changes in 28 (84.8%) subjects as compared to the participants with lesser smoking duration. No significant association was also seen between the severity of exacerbation and ECG changes (Table 3).

**Table 3:** ECG changes in relation to age, gender, smoking duration, and severity of exacerbation Data are presented as n (%). P-value < 0.05 is considered as statistically significant.

ECG Changes	Yes	No	P-value
ECG changes in relation to age			
23-35 years	13 (39.4%)	44 (41.1%)	0.547
36-50 years	9 (27.3%)	37 (34.6%)	
>50 years	11 (33.3%)	26 (24.3%)	
ECG changes in relation to gender			
Male	29 (87.9%)	95 (88.8%)	0.886
Female	4 (12.1%)	12 (11.2%)	
ECG changes in relation to the smoking duration			
4-5 years	5 (15.2%)	35 (32.7%)	0.051
>5 years	28 (84.8%)	72 (67.3%)	
ECG changes in relation to the severity of exacerbation			
Mild	7 (21.2%)	39 (36.4%)	0.249
Moderate	12 (36.4%)	34 (31.8%)	
Severe	14 (42.4%)	34 (31.8%)	

## Discussion

Patients with COPD who presented with severe acute exacerbation and long smoking duration had a high prevalence of ECG abnormalities. Moreover, the prevalence of ECG abnormalities was higher in men and the younger age group.

The frequency of ECG abnormalities increased with pulmonary obstruction severity [10]. In our study, 23.6% of the participants presented with ECG abnormalities, of which the most common are right atrial enlargement and right ventricular hypertrophy. Our results are partly consistent with the findings of Grymonprez et al., which showed that most patients with COPD experience right atrial enlargement and right ventricular hypertrophy [14]. ECG abnormalities are extremely common at baseline in patients with acute COPD exacerbation, as reported by Harvey and Hancox [15]. That is, 8% of patients had ST-segment depression; 37% had T wave changes; 17% had conduction block; 6% had new abnormalities [15]. Moreover, Jatav et al. and Sekhar et al. reported that patients with COPD had a higher prevalence of right ventricular hypertrophy, which is consistent with our study [16,17]. However, our findings were contrasting to those of Singh II et al., which revealed that the frequency of right axis deviation, p-pulmonale, low voltage QRS, and ventricular conduction were higher than that of right atrial enlargement and right ventricular hypertrophy [18].

This research found that ECG abnormalities had male predominance, and this finding is in accordance with that of other studies [19-21]. However, our results were contrasting to those of the study of Alkukhun et al., which was conducted in India. In this report, women with COPD had a high prevalence of ECG abnormalities [22]. Although our results regarding age were not significant, we found that the frequency of ECG abnormalities was high in the young age group (between 23 and 35 years). This result was not consistent with that of recent studies showing that the frequency of ECG abnormalities was high in the old age group (>50 years) [13]. Moreover, there was a high frequency of severe acute exacerbation through the stages from mild to severe, which is similar to the study of Dave et al. [23]. The patients in our study had a longer smoking duration, which is similar to other reports. Hence, approximately 50%-80% of patients with COPD were smokers [24]. ECG abnormalities of better understanding in COPD can help advance the ECG interpretation findings and identify the dominant airway pathophysiology of diseases. Thus, early cardiac screening should be emphasized to help identify prognosis and the risk for morbidity and mortality among patients with COPD.

## Conclusions

Patients with COPD who present with severe acute exacerbation and long smoking duration had a high prevalence of ECG abnormalities. Hence, ECG may be a valuable tool for detecting ischemic abnormalities among patients with COPD, independent of previously known heart disease, in clinical settings.
